# Simultaneous determination of five active compounds in *chimonanthus nitens* by double-development HPTLC and scanning densitometry

**DOI:** 10.1186/1752-153X-6-46

**Published:** 2012-05-22

**Authors:** Bin Zhou, Miao Tan, Jing-feng Lu, Jing Zhao, Ai-feng Xie, Shao-ping Li

**Affiliations:** 1 School of Pharmacy, Jiangxi Science and Technology Normal University, Nanchang, China; 2 State Key Laboratory of Quality Research in Chinese Medicine, and Institute of Chinese Medical Sciences, University of Macau, Macao, SAR, China; 3 Jiangxi Youmei Pharmaceutical Company Ltd, Wuyuan, China

## Abstract

**Background:**

*Chimonanthus nitens* (family Calycanthaceae), Shanlamei in Chinese, is an unique species in China. The extract of dried leaves of *Chimonanthus nitens* has anti-inflammatory, antipyretic and antitussive effects. Terpenes, coumarins, and flavonoids are usually regarded as the main active components. Therefore, simultaneous determination of these compounds is very important to control the quality of *Chimonanthus nitens*.

**Results:**

A double-development TLC method was developed for simultaneous analysis of five compounds in *Chimonanthus nitens*. The chromatography was performed on silica gel 60 plate with chloroform-methanol (9∶1, v/v) and petroleum ether-ethyl acetate (10∶1, v/v) as mobile phase for twice development. Their characteristic TLC profiles were observed under UV light at 365 nm and the bands were then revealed by reaction with 1% vanillin-H_2_SO_4_ solution. Quantification of three monoterpenes was achieved by densitometry at 545 nm (β-caryophyllene) or 606 nm (cineole and linalool). Two coumarins (scopoletin and scoparone) were determined by densitometry at 340 nm with filter wavelength of 370 nm. The investigated compounds had good linearity (*R*^2^ >0.99) within test ranges.

**Conclusions:**

The developed double-development TLC method is helpful to control the quality of *Chimonanthus nitens*, which is simple and accurate.

## Background

The dry leaves of *Chimonanthus nitens* Oliv., an unique species in China, have been extensively used for treating colds and influenza. Pharmacological studies have shown that it has antitussive, anti-inflammatory, antipyretic, antimicrobial, and antihypertensive activities [1,2]. The chemical components in leaves of *Chimonanthus nitens* include volatile oil, coumarins, flavonoids, and alkaloids [3]‐[5]. Especially, the volatile oil, accounting for 1.82-2.46%, is one of kinds of active compounds in leaves of *Chimonanthus nitens*[1], and the coumarins also have definite biological activities [6]. Therefore, simultaneous analysis of these active compounds is very important to ensure the safety and efficacy of *Chimonanthus nitens*. GC-MS analysis showed that terpenes, such as cineole, linalool and β-caryophyllene, were the main ingredients of volatile oil from *Chimonanthus nitens*[7]‐[10].

This study was to establish a method for simultaneous separation of three monoterpenes (linalool, cineole and β-caryophyllene) and two coumarins (scopoletin and scoparone) on a single TLC plate by using double development because of their obvious difference of polarity [11,12], and quantification by scanning densitometry. This method could be used for quality control of *Chimonanthus nitens*.

## Experimental

### Chemicals and materials

Ethyl acetate, petroleum ether and methanol were from Uni-Chem (Belgrade, Serbia and Montenegro). Formic acid was obtained from Guangdong Guanghua Chemical Factory Co., Ltd (Shantou, Guangdong). Deionized water was prepared by Millipore Milli Q-Plus system (Millipore, Billerica, MA, USA). Chloroform was purchased from Unichem (Belgrade, Serbia and Montenegro).

Reference standards of scopoletin and scoparone were purchased from the National Institute for Food and Drug control (Beijing, China), and cineole, β-caryophyllene and linalool were obtained from International Laboratory (Lexington, USA). *Chimonanthus nitens*, obtained from Jiangxi Youmei Pharmaceutical Company Ltd. (Wuyuan, China), was identified, and voucher specimens were deposited at the Institute of Chinese Medical Sciences, University of Macau, Macao, China.

### Standard and sample preparation

The reference compounds were accurately weighed and dissolved in methanol, the mixed standards solution was obtained by mixing the stock solutions. The concentration of five compounds was 0.755 mg·ml^-1^ (cineole), 0.725 mg·ml^-1^ (β-caryophyllene), 1.800 mg·ml^-1^ (linalool), 3.720 μg·ml^-1^ (scoparone) and 5.360 μg·ml^-1^ (scopoletin), respectively.

Dried leaves powder (500 mg) of *Chimonanthus nitens* was mixed with 5 mL methanol in a sealed tube. The solution was treated in an ultrasonic clean bath (881w, 43 kHz, Bransonic, Danbury, CT) for 60 min, at room temperature (25 ± 2°C). Then methanol was added to compensate for the lost weight during the extraction. After centrifugation in an Allegra X-15R refrigerated centrifuge (Beckman Coulter, Fullerton, CA) for 10 min (at 3500 rpm), the supernatant was collected for analysis.

### TLC

Chromatography was performed on Silica gel 60 TLC plates (Merk, Darmstadt, Germany), and a HPTLC system (Desaga GmbH, Germany) including AS30 HPTLC Applicator, CD60 HPTLC densitometer with Pro Quant Windows software. Mixed standards (10 μL) and samples (5 μL) of *Chimonanthus* were spotted in duplicate on the plate as bands 7 mm wide, 8 mm apart and 10 mm from the bottom edge, respectively. First, the plate was developed to a distance of 40 mm with chloroform-methanol (9∶1, v/v) in a Desaga 20 cm × 10 cm glass flat-bottom chamber after equilibration with mobile phase vapor for 10 min. The developed plate was dried in a stream of cool air, and then further developed to the distance of 90 mm with petroleum ether-ethyl acetate (10∶1, v/v) in another chamber after equilibration with the same solution vapor. The developed plate was viewed under UV 365 nm and scanned at 340 nm with filter wavelength of 370 nm for quantitative determination of two coumarins (scopoletin and scoparone). and then colorized with vanillin-H_2_SO_4_ solution (1 g vanillin dissolved in 100 mL 1% H_2_SO_4_) and heated at 85°C on a YOKO-XR plate heater (Wuhan YOKO technology Ltd., China) to make spots colorized clearly. The treated plate covered with a transparent glass and scanned at 545 nm (β-caryophyllene) or 606 nm (cineole and linalool) in reflectance-extinction mode by use of the densitometer. The slit dimensions were 0.02 mm × 4 mm.

### Method validation

For calibration and assessment of linearity, the mixed standard solutions were applied in eight amounts (1, 2, 4, 6, 8, 10, 12 and 15 μL). Linearity was determined by constructing calibration plots of peak area against amounts of each analyte.

To assess stability, the peak area was measured by applying the mixed standards solution, developing the plate, and scanning each band every 5 min for 30 min.

Instrumental precision was checked by scanning the same spot of the investigated compound in mixed standards solution (10 μL) six times. Identical plate precision was determined by analyzing six spots of the mixed standards solution (10 μL) on one plate, while different plate precision was tested by determining one spot of the mixed standards solution (10 μL) on six TLC plates, respectively.

The limits of detection (LOD) and quantification (LOQ) were determined as the amounts for which the signal-to-noise ratios (*S*/*N*) were 3∶1 and 10∶1, respectively. Noise was defined as the peak area corresponding to the blank solution.

Method accuracy was evaluated by measurement of detection recovery and extraction recovery. Detection recovery was tested at three concentrations of mixed standard solution, namely 80% (low), 100% (medium), and 120% (high) of the medium amount of the linear range of the relevant tested compound. Recovery (%) was calculated as *A*_m_/*A*_s_ × 100%, where *A*_m_ was the measured amount and *A*_s_ was the sampling amount of the analyte. To determine extraction recovery, three successive extractions of one sample were carried out to indentify the relevant tested compounds by TLC-densitometry.

The repeatability was evaluated by preparing and analyzing six solutions of the same sample (500 mg each). One spot of each solution was analyzed on the same plate, and RSD of the investigated compounds were calculated.

## Result and discussion

### Optimization of the method

**Figure 1:**
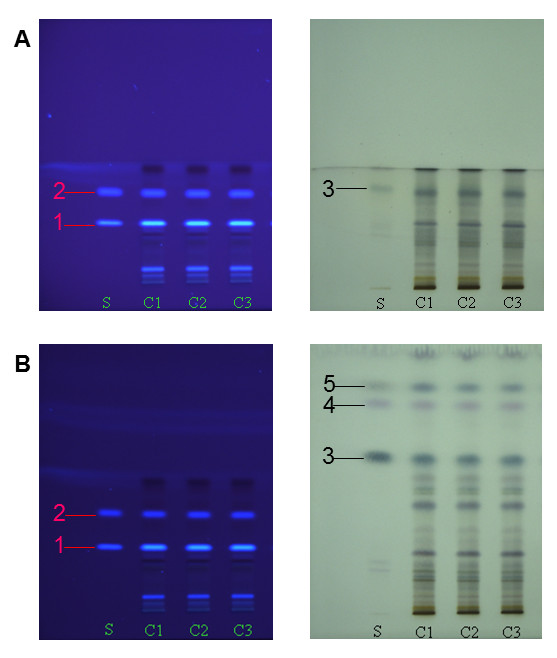
**TLC chromatograms of mixed standards and methanol extracts of*Chimonanthus nitens*for (A) one and (B) twice development on silica gel 60 TLC plate viewed (left) at λ = 365 nm and (right) after coloration with 1% vanillin-H**_**2**_**SO**_**4**_**solution.** Mobile phase of chloroform-methanol (9‐1, v/v) was for the first development, and petroleum ether-ethyl acetate (10‐1, v/v) was for the second development. C1-C3 were three samples of *Chimonanthus nitens* derived from Wuyuan, Jiangxi Province. **S**, mixed standards; **1**, scopoletin; **2**, scoparone; **3**, linalool; **4**, β-caryophyllene; **5**, cineole.

TLC analysis was performed under carefully optimized chromatographic condition. Chloroform-methanol (9∶1, 8∶2, v/v), ethyl acetate-formic acid-water (8∶1∶1, 7∶1.5∶1.5, v/v), and petroleum ether-ethyl acetate (9∶1, 10:1, v/v) were not available for simultaneous separation of low- and high-polarity compounds in *Chimonanthus* with good resolution in one run. Thus, twice development was employed. The optimum development reagents are: chloroform-methanol (9∶1, v/v) for the first development and petroleum ether-ethyl acetate (10∶1, v/v) for the second development. As the results, the high-polarity compounds such as scopoletin (**1**) and scoparone (**2**) were separated, while the low-polarity compounds were developed as one main band in the first run because of the high polarity of mobile phase. Then the low-polarity components, including linalool (**3**), β-caryophyllene (**4**) and cineole (**5**), were further resolved using low-polarity mobile phase in the second run, while the high-polarity compounds could not be driven. Finally, five compounds in *Chimonanthus nitens* were well separated in twice development (Figure 1).

**Figure 2:**
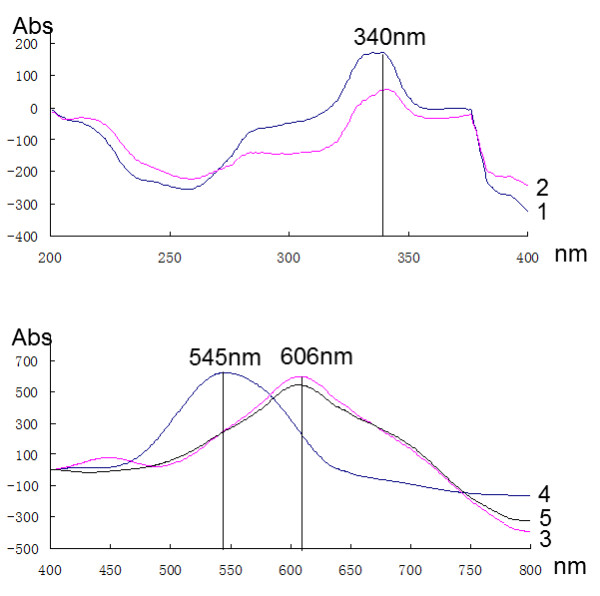
**Vis/UV spectra of investigated compounds.1**, scopoletin; **2**, scoparone; **3**, linalool; **4**, β-caryophyllene; **5**, cineole.

The detection wavelengths for quantification of the tested compounds were selected based on their spectra (Figure 2). The detection wavelength for determination of two coumarins, scopoletin (**1**) and scoparone (**2**), was set as UV 340 nm with filter wavelength of 370 nm before colouration, while the quantification of β-caryophyllene (**4**) was chosen at 545 nm, as well as linalool (**3**) and cineole (**5**) were at 606 nm after colouration.

### Method validation

Regression, precision and stability for the analyes were determined and summarized in Table 1. All plots were linear (*R*^*2*^ > 0.99) within the ranges examined. The stability results shows that although peak area of each investigated compound decreased as time passed, it was stable within 30 min. Instrumental precision (RSD, %) was less than 5% (n = 6). For identical plates, precision as overall RSD was below 4% (n = 6), and the different plates precision was also determined (Table 1). LOD and LOQ for the tested compounds were listed in Table 2. Good accuracy, detection recovery and extraction recovery, were achieved for the analytes (Table 2). The repeatability for all the analytes was also shown in Table 2.

**Table 1:** **Summary for the tested samples of*Chimonanthus nitens***

**Analyte**	**Regression**			**Precision (RSD%,n = 6)**			**Stability** **(RSD, %)**
	**Linear regression ** **equation**	**Linear range [μg]**	***R***^**2**^	**Instrument**	**Identical plate**	**Different ** **plate**	
Linalool	Y = 125.1 x + 368.8	1.80-18.00	0.990	0.6	3.5	9.7	2.0
β-caryophyllene	Y = 356.7 x + 195.7	0.72-10.87	0.992	1.5	8.0	9.0	3.0
cineole	Y = 206.2 x + 199.9	0.76-11.40	0.991	4.8	4.0	4.3	3.3
scopoletin	Y = 45.2 x + 45.5	0.011-0.064	0.996	1.2	2.6	5.4	4.2
scoparone	Y = 48.7 x + 236.4	0.015-0.045	0.993	1.8	3.7	8.3	2.5

**Table 2:** LOD, LOQ, repeatability, and accuracy for the compounds investigated

**Analyte**	**LOD** **[ng]**	**LOQ** **[ng]**	**Repeatability** **(RSD,%,n = 6)**	**Accuracy (%)**			
				**Extraction recovery** **(RSD,%, n = 3)**	**Detection recovery (RSD%, n = 3)**		
					**Low**	**Medium**	**High**
Linalool	35	87	3.2	96.3 (2.5)	109.8 (1.9)	95.8 (2.9)	97.5 (2.0)
β-caryophyllene	28	54	4.4	89.7 (3.8)	96.7 (3.6)	90.7 (2.2)	86.1 (3.3)
cineole	9	43	2.7	92.8 (1.6)	82.3 (2.7)	93.4 (4.0)	87.6 (4.1)
scopoletin	2	6	2.9	95.1 (2.4)	88.7(2.2)	106.0(4.0)	92.3(3.5)
scoparone	1	3	4.8	94.6 (1.9)	102.0(3.7)	90.4(2.9)	97.8(3.7)

### Analysis of the compounds in chimonanthus nitens

**Figure 3:**
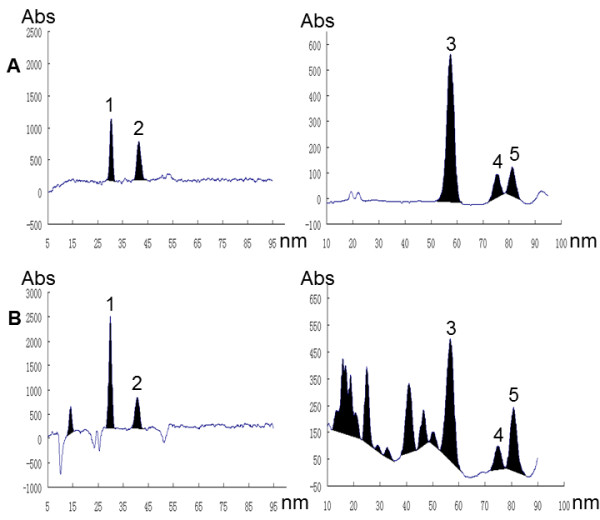
**Typical twice development TLC densitograms of (A) mixed standards and (B) methanol extracts of*chimonanthus nitens*detected at (left) 340 nm using filter of 370 nm and (right) 606 nm after coloration of 1% vanillin-H**_**2**_**SO**_**4**_**solution.1**, scopoletin; **2**,scoparone; **3**, linalool; **4**, β-caryophyllene; **5**, cineole.

Quantitative analysis of *Chimonanthus nitens* samples collected from Wuyuan (Jiangxi, China) was performed by densitometric TLC. The typical TLC scanning profiles based on Figure 1 were shown in Figure 3. The amounts of five investigated compounds in *Chimonanthus nitens* were (n = 3): 0.11 ± 0.01 mg·g^-1^, 0.05 ± 0.01 mg·g^-1^, 3.47 ± 0.24 mg·g^-1^, 0.53 ± 0.03 mg·g^-1^ and 0.61 ± 0.01 mg·g^-1^ for scopoletin, scoparone, linalool, β-caryophyllene and cineole, respectively.

## Conclusion

A double-development TLC method was developed for simultaneous analysis of five compounds, including three monoterpenes (linalool, β-caryophyllene and cineole), two coumarins (scopoletin and scoparone), in *Chimonanthus nitens*, which was helpful to control its quality.

## Abbreviations

TLC, Thin layer chromatography; GC-MS, Gas chromatography–mass spectrometry; HPLC, High performance liquid chromatography.

## Authors’ contributions

SPL initiated and designed the study. The extraction and method developments were conducted by BZ who drafted the manuscript, and all other authors. All authors contributed to data analysis and manuscript finalization.
